# Diabetic nephropathy patients show hyper-responsiveness to N6-carboxymethyllysine

**DOI:** 10.1590/1414-431X2022e11984

**Published:** 2022-07-25

**Authors:** C.G. Dias, L. Venkataswamy, S. Balakrishna

**Affiliations:** 1 Department of Cell Biology and Molecular Genetics, Sri Devaraj Urs Academy of Higher Education and Research, Kolar, India; 2 Department of General Medicine, Sri Devaraj Urs Medical College, Sri Devaraj Urs Academy of Higher Education and Research, Kolar, India

**Keywords:** Diabetic nephropathy, Receptor for advanced glycation end-product pathway, N6-carboxymethyllysine, Nuclear factor-kappa B, Tumor necrosis factor

## Abstract

The aim of this study was to evaluate the impact of N6-carboxymethyllysine (CML) on *NF-κB* gene expression and tumor necrosis factor (TNF) production in diabetic nephropathy. This was an observational study comprised of three groups: diabetic nephropathy (n=30), type II diabetes mellitus (n=28), and healthy volunteers (n=30). Blood samples collected from the study participants were cultured for 24 h in the presence of CML or an appropriate control. After incubation, the cultures were centrifuged to separate the cells from the conditioned media. cDNA was prepared from the cell pellet and used to quantify *NF-κB* gene expression by quantitative real-time polymerase chain reaction (PCR). The conditioned media were used to measure TNF production by enzyme-linked immunosorbent assay (ELISA). The CML-induced fold change in *NF-κB* gene expression was significantly different among the study groups (P=5.4×10^-5^). Also, the CML-induced fold change in TNF levels was significantly different among the three groups (P=4.3×10^-8^). These results imply that patients with diabetic nephropathy and type II diabetes mellitus showed an elevated response to CML.

## Introduction

Type II diabetes mellitus (T2DM) is a major public health burden that affects almost 463 million people worldwide (1). Elevated blood sugar levels can cause damage to multiple organ systems, as it mainly affects the vasculature. A major problem of T2DM is that it progresses into secondary complications, such as macrovascular complications (cardio- and cerebrovascular disease) and microvascular complications (retinopathy, nephropathy, and peripheral neuropathy) (2), of which nephropathy stands first in terms of the prevalence of microvascular complications (3).

In a subset of patients, T2DM eventually leads to renal damage, a condition referred to as diabetic nephropathy (DN) (4). DN eventually progresses into end-stage renal disease, which is a fatal condition that contributes to the increased mortality of individuals with diabetes (5). Therefore, there is an urgent need to uncover the mechanism involved in the progression of T2DM into DN.

DN involves histological changes in the nephron, such as basement membrane expansion, tubulointerstitial fibrosis, glomerulosclerosis, and podocytopathy (6). Inflammation plays an important role in the pathogenesis of DN. Abnormal inflammation in DN is linked to advanced glycation end-products (AGEs). These substances are formed due to non-enzymatic glycation of proteins and lipids followed by oxidation (7). AGE levels are elevated in T2DM patients due to hyperglycemia (8-10). AGEs are potent activators of inflammation through the receptor for advanced glycation end-product (RAGE) signaling pathway. The RAGE receptor is expressed in several types of cells, such as endothelial cells, smooth muscle cells, mononuclear phagocytes, neurons, and cardiac myocytes (10). Activation of RAGE by AGE substances elicits a signaling cascade that eventually leads to the activation of a transcription factor called nuclear factor kappa B (NF-κB) (11). NF-κB promotes the expression of various proinflammatory cytokines that, when released into the microenvironment, cause inflammation (12).

There is limited information on the functional status of the RAGE signaling pathway in DN. The RAGE pathway may produce excessive inflammatory signaling for two reasons. First, the potent activator of the pathway, specifically AGEs, is known to be elevated in DN (13). Second, the RAGE pathway is abnormally hyper-responsive to AGE substances. There is no evidence in this direction. Therefore, we tested this hypothesis by evaluating the AGE-induced fold change in the key mediators and effectors of the RAGE pathway.

N6-carboxymethyllysine (CML) was used as the AGE representative since it is the predominant AGE formed in T2DM patients. Elevated levels of CML were found in the serum and organs (such as kidney) of diabetic patients (14). Abnormally high levels of circulating CML and their accumulation in tissues are thought to represent a critical step in the pathogenesis of DN (15).

## Material and Methods

### Study design and participant selection

This was a comparative study comprised of three groups. Group 1 included patients diagnosed with DN. Group 2 consisted of patients diagnosed with T2DM, but without nephropathy. Group 3 was comprised of healthy volunteers. The study participants were recruited from R.L. Jalappa Hospital and Research Centre, Kolar, India, from January 2019 to April 2020. The study was approved by the Institutional Ethics Committee of Sri Devaraj Urs Medical College, Kolar, Karnataka, India. Written informed consent was obtained before participant recruitment. The inclusion criteria adopted when selecting the participants for each group are given in Table 1. Staging of nephropathy was carried out according to the method of Haneda et al. (16).

**Table 1:** Patient selection criteria.

Criteria	Diabetic nephropathy	Type II diabetes	Healthy volunteers
Inclusion	a) Stages 4 and 5* b) Fasting plasma glucose(FPG>126 mg/dL) c) Glycated hemoglobin(HbA1c>6.5%) d) Creatinine (>1.2 mg/dL) e) Estimated glomerular filtration rate (eGFR<125 mL/min 180 L/day and2 mL/s) f) Blood urea nitrogen(BUN>24 mg/dL)	a) Fasting plasma glucose (>126 mg/dL) b) Glycated hemoglobin (>6.5%) c) Creatinine (<1.2 mg/dL)	a) Age- and gender-matched individuals b) Fasting plasma glucose(<100 mg/dL)
Exclusion	a) Stages 1 to 3* b) Chronic co-morbidities	a) Microvascular complications	a) No history of chronic illness

* Staging of nephropathy was carried out according to the method of Haneda et al. (16).

### Blood culture

Fresh blood samples (3 mL) were collected from the study participants in a sterile EDTA vacutainer and subsequently used for the cell culture experiment. Whole blood, as opposed to peripheral blood mononuclear cells (PBMCs), was used as it shows a robust cytokine response (17). The cultures were set up by mixing 500 µL of whole blood with 4.5 mL of RPMI-1640 medium (supplemented with 10% fetal bovine serum and 1% penicillin-streptomycin). The cultures were incubated at 37°C for 24 h in a 5% CO_2_ atmosphere. Two cultures were set up for each sample. The first culture was treated with CML (Cat #14580; Sigma Aldrich Co., USA) to a final concentration of 10 µM (18). The second culture was treated with phosphate buffered saline (vehicle control). The experiments were conducted in duplicate.

### Transcript preparation

At the end of the incubation period, the blood cultures were centrifuged at 1500 *g* for 10 min at room temperature, and the supernatant of the conditioned media was stored at -80°C in aliquots. The cell pellet was used to isolate total RNA via the Trizol method using a commercial kit (Cat #15596018; Thermo Fisher Scientific, USA). Total RNA (0.5 µg) was then used to prepare cDNA using a commercial kit (Cat #1708891; iScript™ cDNA synthesis kit; Bio-Rad Laboratories, USA). cDNA samples were stored at -20°C until subsequent analysis.

### Gene expression analysis

The comparative threshold cycle (Ct) method was used to quantify the relative gene expression normalized to the housekeeping gene, *GAPDH*. mRNA levels were measured by quantitative real-time polymerase chain reaction (PCR) using the SYBR green method (Cat #1725271; SsoAdvanced Universal SYBR Green; Bio-Rad Laboratories). The following primer pair was used for GAPDH gene expression: 5′-GATCATCAGCAATGCCTCCT-3′ and 3′-GACTGTGGTCATGAGTCCTTC-5′, for the sequence, NM_001289745.3. The thermal cycling program for *GAPDH* gene expression involved initial denaturation at 95°C for 3 min, followed by 39 cycles at 95°C for 10 s, and 55°C for 30 s. *NF-κB* gene expression for the sequence NM_001077494.3 was analyzed using the following primer pair: 5′-TACCGACAGACAACCTCACC-3′ and 3′-CAGCTTGTCTCGGGTTTCTG-5′. The thermal cycling program for *NF-κB* gene expression consisted of initial denaturation at 95°C for 3 min, followed by 39 cycles at 95°C for 10 s, and 62.4°C for 30 s. The fold change in *NF-kB* gene expression was determined by calculating 2^-ΔΔCT^, where ΔCT = Ct (*NF-κB*) - Ct (*GAPDH*) and ΔΔCT = ΔCt (treated) - ΔCt (untreated). The ΔCt values were used for the statistical comparisons between treated and untreated samples within each study group. The ΔΔCT values were used for statistical comparisons between study groups. The assay was conducted in duplicate. A positive control reaction for each gene (pooled and aliquoted cDNA) was included to adjust for inter-run variability. The variability of the positive control was <0.1 Ct within the plate and <0.3 Ct between plates.

### Estimation of TNF levels

Tumor necrosis factor (TNF) levels were measured in the conditioned media by enzyme-linked immunosorbent assay (ELISA) using a commercially available kit (Cat #SEA133Hu; Cloud-Clone Corp., USA).

### Statistical analysis

Statistical analysis was carried out using SPSS Statistics V20 (IBM Corporation, USA). To verify whether the data were normally distributed, the Shapiro-Wilk test was performed with Q-Q and normality plots. Means and standard deviations were determined if the data showed a normal distribution; otherwise, the median and interquartile range (IQR) were calculated. Parametric tests were used to compare data showing a normal distribution and non-parametric tests were used for data that did not follow a normal distribution. A P-value less than 0.05 was considered statistically significant.

## Results

### Clinical profile of the study participants

A total of 88 participants were included in the study, 30 of whom had DN, 28 had T2DM, and 30 were healthy controls. The clinical and demographic profiles of the study participants are summarized in Table 2.

**Table 2:** Clinical and demographic profile of the study participants.

Parameters	Diabetic nephropathy (n=30)	Type II diabetes (n=28)	Healthy volunteers (n=30)
Age (years)	59.9±7.7	54.9±7.5	55.0±7.5
Gender (male/female, %)	44.4/55.5	45.4/54.5	46.9/53.1
Fasting plasma glucose (mg/dL)	150.2±23.3	152.1±19.9	77.0±8.3
HbA1_C_(%)	6.9±0.8	6.9±0.7	4.3±0.3
Serum creatinine (mg/dL)	3.7±0.2	0.9±0.3	0.62±0.2
eGFR (mL·min^-1^·(1.73 m^3^)^-1^)	15.5±6.3	97.2±4.6	77.9±9.2
Blood urea nitrogen (mg/dL)	49.5 ±16.4	18.46±1.65	16.01±1.44

Data are reported as means±SD, except for gender.

### Effect of CML on *NF-&mac_kgr;B* gene expression

**Figure 1:**
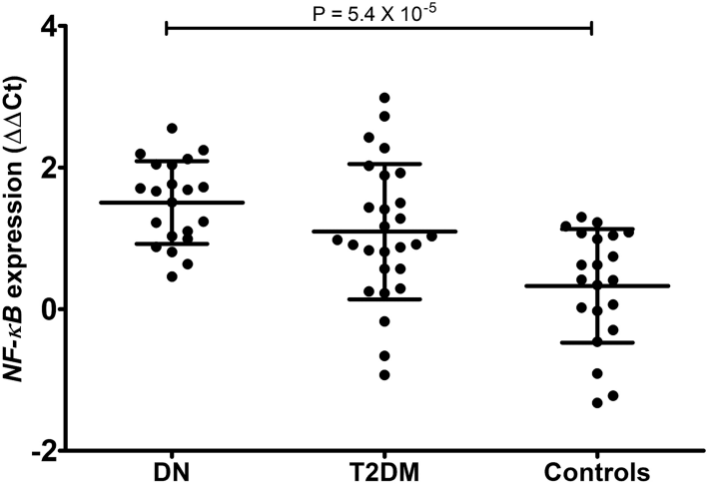
Comparison of N6-carboxymethyllysine (CML)-induced fold change in *NF-κB* gene expression between the study groups. Data are reported as mean and standard deviation (ANOVA). DN: diabetic nephropathy; T2DM: type II diabetes mellitus.

**Figure 2:**
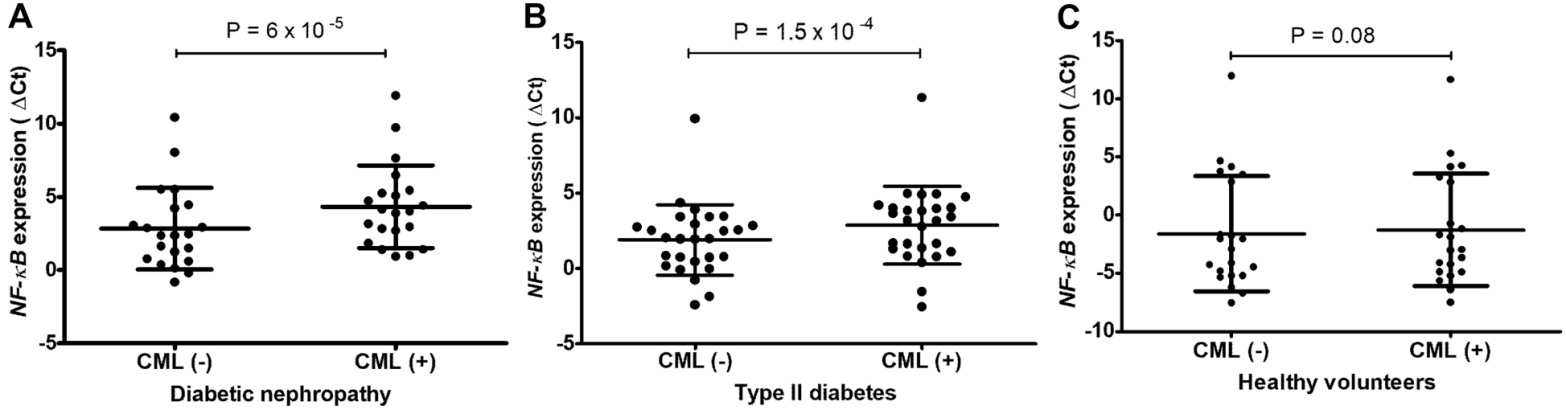
Effect of N6-carboxymethyllysine (CML) on *NF-κB* gene expression in the study groups. Normalized gene expression in **A**, diabetic nephropathy; **B**, type II diabetes; and **C**, healthy volunteers. Data are reported as median and interquartile range (Wilcoxon signed-rank test).

First, we compared the effect of CML treatment on *NF-κB* gene expression in each study group. The ΔCt values did not show a normal distribution. Therefore, the median and IQR were calculated, and non-parametric tests were used for comparison. We compared the relative expression (ΔΔCt) of the *NF-κB* gene between the three study groups. The ΔΔCt values showed a normal distribution. Therefore, the mean and standard deviation were calculated, and parametric tests were used for comparison. ΔΔCt was significantly different among the three groups (P=5.4×10^-5^; one-way analysis of variance [ANOVA]). The results are shown in Figure 1. Second, CML treatment resulted in higher normalized expression (ΔCt) of the *NF-κB* gene in DN (P=6.0×10^-5^) and T2DM (P=1.5×10^-5^), but not in healthy volunteers (P=0.08) (Wilcoxon signed-rank test). The results are shown in Figure 2. The CML-induced fold change in *NF-κB* expression was 2.8 (0.95CI: 2.5-3.62) in DN and 2.1 (0.95CI: 1.91-3.34) in T2DM.

These results indicate that CML treatment upregulated the expression of the *NF-κB* gene in DN and T2DM, but not in healthy volunteers.

### Effect of CML on TNF production

The TNF levels in all the study groups followed a normal distribution. Therefore, mean and standard deviation were calculated, and parametric tests were used for comparison.

**Figure 3:**
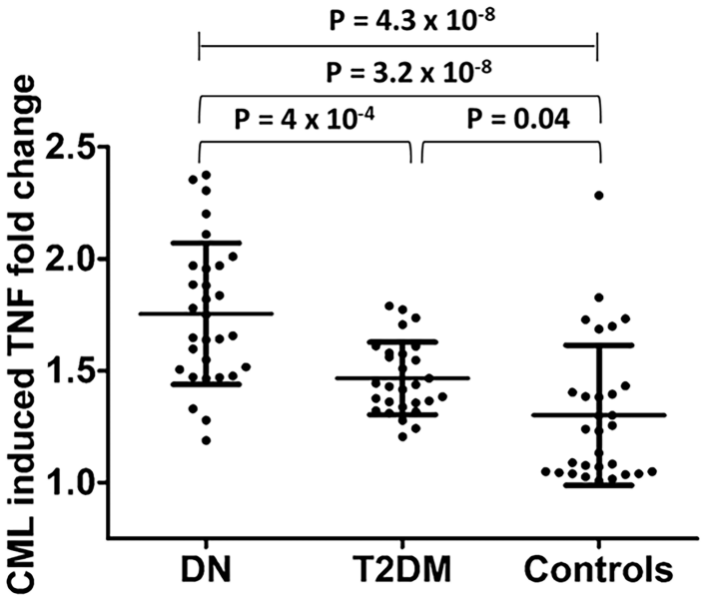
N6-carboxymethyllysine (CML)-induced tumor necrosis factor (TNF) fold change among the study groups. Data are reported as mean and standard deviation (ANOVA). DN: diabetic nephropathy; T2DM: type II diabetes mellitus.

Firstly, we compared the fold change in CML-induced TNF production between the three study groups. A significant difference was observed between the three study groups (P=4.3×10^-8^; one-way ANOVA). The results are shown in Figure 3.

**Figure 4:**
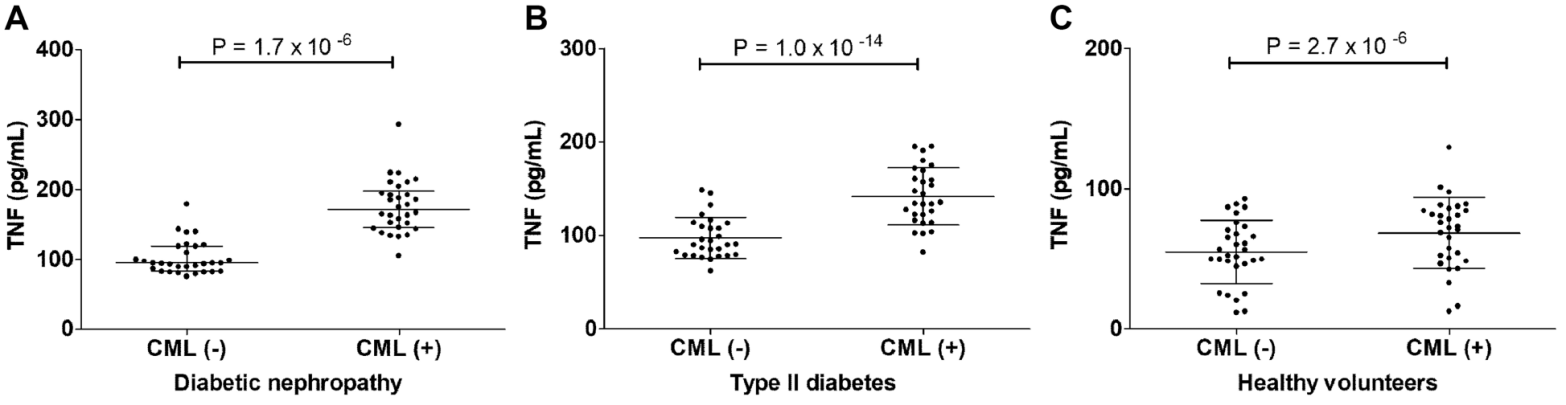
Effect of N6-carboxymethyllysine (CML) on TNF production in the study groups. TNF levels in the treated and untreated conditioned media in **A**, diabetic nephropathy; **B**, type II diabetes; and **C**, healthy volunteers. Data are reported as mean and standard deviation (paired *t*-test).

Secondly, we compared the effect of CML treatment on TNF production in each study group. CML treatment resulted in elevated TNF production in all three study groups, with the highest increase observed in the DN group. The average fold increase was 1.76±0.32 (P=1.7×10^-6^; paired *t*-test) in DN, 1.47±0.16 (P=1.0×10^-14^; paired *t*-test) in T2DM, and 1.30±0.31 (P=2.7×10^-6^; paired *t*-test) in the healthy volunteers. The results are shown in Figure 4.

These results indicate that CML treatment enhanced TNF production in DN and T2DM compared with healthy volunteers.

## Discussion

The purpose of this study was to compare the effect of CML treatment on the expression of key mediators in the RAGE pathway in the blood cells of patients with nephropathy and those with T2DM.

This study was carried out using blood cells, as it is not possible to perform renal biopsy for ethical reasons. Blood cells were used as a surrogate because these cells also express RAGE. In addition, the aim of this study was to compare the relative responsiveness (fold change) of the RAGE pathway to an inducer in the three groups. Tissue-specific differences in RAGE expression in blood and renal cells may affect total responsiveness, but these differences are unlikely to affect the relative responsiveness between groups. The outcome measure for this study was based on the products that result from the activation of the RAGE-dependent inflammatory pathway.

The main findings of this study are that CML treatment resulted in: i) significant upregulation of the *NF-κB* gene expression in DN and T2DM; and ii) significantly higher levels of TNF production in DN and T2DM. These results indicated that patients with DN and T2DM showed hyper-responsiveness to CML.

Previous studies have shown that inflammatory markers are elevated in DN. Higher levels of *NF-κB* mRNA have been reported in the PBMCs, renal biopsy results, and urine of patients with DN (19-21). Furthermore, an increase in the protein and mRNA levels of *NF-κB* in PBMCs has been linked to increasing severity stages of DN (22). Several studies have reported elevated levels of serum TNF in DN patients and T2DM. In addition, the relationship was further confirmed in a meta-analysis (23). However, there is limited information on the mechanisms underlying the elevated inflammatory markers in DN and T2DM.

The results of this study indicated that the elevation of inflammatory markers in DN and T2DM may arise due to a hyper-responsiveness to AGE. Genetic variations may be responsible for this hyper-responsiveness. Single nucleotide polymorphisms in the promoter region of the *NF-κB* and *TNF* genes are reportedly associated with DN and T2DM (24,25). Genetic variations in the key genes of the RAGE signaling pathway might constitute a predisposing factor that can lead to an abnormal inflammatory response when triggered by AGE.

There are several limitations to this study. First, the RAGE pathway carries out an elaborate process that involves several mediators and effectors (KEGG pathway). We have not quantified the level of RAGE or the other cytokines involved in the pathway; for instance, interleukin (IL)-1β, IL-6, IL-8, CD36, and MCP-1 were not measured (26). Also, we did not examine the other transcription factor in the pathway, AP-1. This may be why increased TNF was observed in healthy volunteers, despite the fact that the *NF-κB* gene expression remained unaltered.

Overall, this study shows that patients with DN and T2DM had an elevated inflammatory response as a result of hyper-responsiveness to AGE. This implies that the AGE-RAGE pathway could be explored as a potential target for ameliorating the development of nephropathy in T2DM patients. Compounds that inhibit the RAGE pathway could reduce AGE-induced inflammation and renal injury. Renal damage represents one of the major concerns in diabetes management. This study provides the conceptual framework for controlling the RAGE pathway in order to prevent the development of renal complications in diabetes patients.
